# Adolescent Mental Health Resilience and Combinations of Caregiver Monitoring and Warmth: A Person-centred Perspective

**DOI:** 10.1007/s10826-022-02287-0

**Published:** 2022-03-26

**Authors:** Linda Theron, Sebastiaan Rothmann, Alexander Makhnach, Michael Ungar

**Affiliations:** 1 Department of Educational Psychology, University of Pretoria, Pretoria, South Africa; 2 Optentia Research Unit, North-West University, Vanderbijlpark, South Africa; 3 Institute of Psychology of the Russian Academy of Sciences, Moscow, Russia; 4 Resilience Research Centre, Dalhousie University, Halifax, NS Canada

**Keywords:** Adolescents, Conduct problems, Depression, Mental health resilience, Parenting, South Africa

## Abstract

Caregiver monitoring and warmth have protective mental health effects for adolescents, including vulnerable adolescents. However, combinations of the aforesaid parenting behaviours and their relationship with adolescent mental health are underexplored, especially among younger and older South African (SA) adolescents challenged by structural disadvantage. Hence, the purpose of this study was to investigate unique profiles of caregiver monitoring and warmth and their associations with depression and conduct problems as reported by younger and older adolescents from disadvantaged SA communities. Latent profile and linear regression analyses were used to examine cross-sectional survey data generated by 891 adolescents from two disadvantaged SA communities (62.2% aged 13–17 [average age: 16.13]; 37.5% aged 18–24 [average age: 20.62]). Two profiles emerged. The first, i.e. substantial caregiver warmth and some monitoring, was associated with younger and older adolescent reports of statistically significantly fewer symptoms of depression and conduct problems. The second, i.e. caregiver monitoring without much warmth, was associated with significantly more symptoms of depression or conduct problems among younger and older adolescents. Traditional gender effects (i.e. higher depression symptoms among girls; higher conduct problem symptoms among boys) were amplified when caregiver monitoring was combined with low warmth. In short, protecting the mental health of younger and older adolescents from disadvantaged communities requires higher levels of caregiver warmth combined with moderate levels of caregiver supervision. Because stressors associated with disadvantaged communities jeopardise warm parenting, supporting caregiver resilience to those stressors is integral to supporting adolescent mental health.

## Highlights

- Considerable caregiver warmth combined with moderate caregiver supervision protected the mental health of adolescents from disadvantaged study communities.
- This protective association held for younger (13–17 years) *and* older (18–24 years) adolescents.
- Most caregivers from the disadvantaged study communities parented warmly; capacity for warm parenting in the face of disadvantage must be acknowledged and sustained.

As in other parts of the world, mental illness poses a serious challenge to the development and functioning of South Africa’s adolescents (Babatunde et al., 2020). While there are no national estimates (Shung-King et al., 2019), internalising and externalising disorder incidence among South African (SA) adolescents (e.g., 13–15% for depression; Eyal & Burns, 2019) reflects international rates (Polanczyk et al., 2015). Still, most SA adolescents have no/limited access to mental health services (Babatunde et al., 2020). Given this paucity, it is important to investigate informal resources – such as parenting factors – that might enable SA adolescents’ mental health resilience (Mthiyane et al., 2021, Shung-King et al., 2019). This article responds to that agenda.

In specific, it reports a person-centred investigation of the association between combinations of parenting behaviours (i.e., caregiver warmth; caregiver monitoring) and symptoms of depression and conduct problems as reported by SA adolescents (*n* = 891) exposed to significant stress (i.e., chronic structural disadvantage). While this investigation fits seminal understandings of the importance of caregiver warmth and monitoring to children’s developmental outcomes (Baumrind, 1971; 2005), it tests their applicability to younger and older adolescents living in South Africa. African adolescents and older adolescents (i.e., 18–24-year-olds; see Sawyer et al., 2018) are under-represented in studies that associate parenting behaviours with adolescent mental health. Given the tendency of many adolescents to live with their parents into early adulthood (Sawyer et al., 2018), it is important to include older adolescents in studies of the relationship between parenting and adolescent mental health. Moreover, the substantive size of the African adolescent population and related predictions of this population’s exponential growth direct urgent attention to the health and wellbeing of African adolescents (Hajjar, 2020).

The results have implications for practitioners wishing to champion the mental health resilience of African adolescents. Unlike typical studies of parenting and adolescent mental health, the results are inclusive of younger and older adolescents. Given the context of the study that this article reports, the results are likely also generalisable to adolescents living in communities challenged by socioeconomic disadvantage and poor service delivery.

## Parenting and Adolescent Mental Health Resilience

In the face of significant stress, adolescent capacity for mental health resilience often relates to how adolescents are parented (Masten, 2014; Masten & Palmer, 2019). Quality parenting is characteristically explained in terms of support (or warmth) and control (Baumrind, 1971; 2005; Hoeve et al., 2009), more specifically behavioural control (i.e., monitoring what adolescents are doing and/or whom they are with, particularly when they are not at home). Behavioural control often requires adolescent willingness to disclose their movements, associations, or plans (Kerr & Stattin, 2000).

Baumrind’s (1971, 2005) classic conceptualisation of parenting styles – i.e., authoritative, authoritarian, permissive, or uninvolved – speaks to varying degrees of caregiver warmth or responsiveness and caregiver monitoring or demandingness. In Baumrind’s early studies, higher responsiveness and moderate demandingness were associated with the most positive developmental outcomes for pre-schoolers (Baumrind, 1971). A follow-up study with these same children when they were age 15, showed that high caregiver control/monitoring and low warmth were associated with the least positive developmental outcomes (Baumrind, 2005). Subsequent studies confirmed that greater caregiver warmth and moderate monitoring were associated with better child and adolescent mental health (Lansford et al., 2018; Logan et al., 2019; Pinquart, 2017; Rothenberg et al., 2020a, b, c; Shuey & Leventhal, 2019). North American children were typically represented in these studies, but Lansford et al., 2018 and Rothenberg and colleagues (2020a, b, c) also included children aged 8 to 13 from China, Colombia, Italy, Jordan, Kenya, the Philippines, Sweden, and Thailand.

Very few studies have investigated combinations of caregiver warmth and monitoring, and their association with the mental health of older adolescents. An exception is the study by Logan et al., 2019 with young men (aged 15–20) from Buffalo, USA; it accentuated specific combinations of caregiver warmth and monitoring and their associations with young men’s mental health. For instance, young men from disadvantaged American neighbourhoods reported better mental health outcomes when they experienced high levels of supportive parenting and moderate monitoring. Those who reported a combination of low supportive parenting, low monitoring and considerable abusive caregiving reported poorer mental health (Logan et al., 2019).

While warm parenting has broad mental health value across diverse cultures, monitoring is likely to be associated with fewer externalising difficulties in sociocultural contexts that de-emphasise adolescent independence (Rothenberg et al., 2020c). Monitoring, which mostly has modest mental health value (Hoeve et al., 2009, Pinquart, 2017), is particularly useful in early and middle adolescence when risk behaviours are often initiated (Bhana et al., 2016, Murphy et al., 2009), and/or in communities characterised by greater disadvantage and instability (Shuey & Leventhal, 2019). While contexts that place children at risk for conduct problems typically elicit caregiver monitoring behaviours, caregiver warmth can also discourage children from engaging in risky behaviours, such as rule breaking, especially in contexts where warm parenting is normative (Rothenberg et al., 2020b). Context can also impact parenting behaviours when it exposes caregivers to significant stress, such as socioeconomic disadvantage, community instability, or violence. Such stresses have strong potential to undermine caregivers’ health and wellbeing and constrain their capacity for responsive, supportive, and enabling parenting (Conger & Donnellan, 2007; Shuey & Leventhal, 2019).

Parenting behaviours can also be shaped by children’s behaviours. Pinquart’s (2017) meta-analysis of 1435 studies of how parenting behaviours relate to child and adolescent symptoms of conduct problems and other externalising difficulties showed bidirectionality effects. Similarly, Lansford et al., 2018 reported that externalising and internalising behaviour difficulties, evidenced by children aged 8 to 13 and living in nine diverse countries, elicited more caregiver control and less caregiver warmth respectively. Put differently, levels of caregiver warmth and control can be child-driven. Rothenburg and colleagues (2020a) reported that such child-driven effects were more probable in sociocultural contexts where externalising and internalising behaviour difficulties among children were rarely reported and/or decried.

## Studies of Parenting and SA Adolescent Mental Health Resilience

SA mental health researchers have been quite neglectful of the association between parenting behaviours and SA adolescents’ internalising and externalising mental health. As summarised next, the exceptions to the aforesaid are published studies with adolescents who are HIV-infected or -affected.

Bhana et al., 2016 related higher levels of caregiver supervision to fewer depression symptoms among 177 isiZulu-speaking, perinatally-infected adolescents (aged 9–14). Shenderovich et al., 2021 reported similar results from their study with 926 HIV-infected, Eastern Cape adolescents (aged 10–19). Using three time-points, they considered whether changes in the adolescent-caregiver relationship corresponded with changes in adolescent depression symptoms and found that higher levels of supervision and supportive parenting generally correlated with lower levels of depression. Similarly, a study with 1060 HIV-positive adolescents (aged 10—19) from the Eastern Cape showed that positive (i.e., warm) parenting was associated with better mental health, including decreased depression (Boyes et al., 2019). Comparable benefits were found for 2477 HIV-affected 10-17-year-olds living in Kwazulu-Natal: caregiving characterised by greater levels of social support was associated with fewer emotional difficulties among the adolescent participants (Casale et al., 2015). Likewise, a Western Cape study with AIDS-orphans (11–24; mean age: 17) showed that these adolescents were likely to report depression symptoms when they had a caregiver that was not supportive (Sharer et al., 2015).

Two of the abovementioned studies (i.e., Boyes et al., 2019; Casale et al., 2015) also reported that supportive caregiving and/or monitoring were associated with decreased externalising symptoms. In addition, Freeze et al. (2014) compared the parenting of 40 adolescent boys (aged 13–17) who were diagnosed with conduct problems and 40 who were not. They found that low parental warmth (particularly by the mother) and high behavioural control (particularly by the father) were associated with conduct problems.

In summary, the evidence of associations between caregiver warmth and/or monitoring and adolescent mental health in South Africa is largely limited to studies with HIV-infected or -affected adolescents (generally younger than 18), and mostly excludes symptoms of externalising difficulties, such as conduct problems. Whereas resilience-enabling parenting typically combines positive parenting behaviours (Logan et al., 2019; Masten, 2014; Masten & Palmer, 2019), the above-mentioned SA studies have also not considered the effects of varied combinations of warmth and monitoring. They preferred variable-centred approaches that assume some degree of homogeneity and are not optimally suited to identifying complex or unique relationships between variables of interest (Meyer & Morin, 2016).

## The Current Study

In South Africa, most adolescents from disadvantaged communities are without access to mental health services and continue to live with their parents or other caregivers as they complete their education and/or search for employment (Shung-King et al., 2019). It is unclear what combination of caregiver warmth and monitoring – if any – will be protective of these adolescents’ mental health, also when adolescents are older (i.e., 18-24-year-olds; see Sawyer et al., 2018). Thus, the current study used a person-centred approach to investigate the possibility of unique profiles of caregiver warmth and monitoring, and related associations with adolescent mental health, as reported by 891 adolescents (aged 14–24) living in disadvantaged SA communities. Following Ahlborg et al. (2019) and Meyer and Morin (2016), we assumed that a person-centred approach could identify complex relationships between caregiver monitoring and warmth and allow exploration of “the underpinnings of unexpected or inconsistent variable-centred associations” (Caesens et al., 2020, p. 691). We held no a priori assumptions for any profile, other than those relating to traditional gender effects reported for internalising and externalising disorders. As such, we assumed that we would find elevated levels of depression symptoms among adolescent girls (Salk et al., 2017) and elevated levels of conduct problem symptoms among adolescent boys (Eme, 2007).

## Method

The data reported are from the Resilient Youth in Stressed Environments (RYSE) study. RYSE investigates what enables and/or constrains the resilience of adolescents in Canadian and SA communities that are stressed by economic and environmental disruptions associated with the oil-and-gas (O&G) industry (Ungar et al., 2021). The economic volatility of the O&G industry is associated with financial precarity for its workforce, with knock-on effects for local businesses and their employees. Similarly, the industry attracts migrant workers; this is frequently associated with a lack of social cohesion, interpersonal conflict/violence, and psychological distress. Often, O&G industry-related stressors jeopardize family functioning and positive parenting (Holtge et al., 2021).

Given this article’s intention to foreground the value of parenting practices to the mental health of African adolescents, it reports SA data only. The SA RYSE sites were semi-urban, O&G-impacted communities in municipalities characterised by environmental degradation, violent crime, indigence, and poor service delivery. In SA, such municipal disadvantage is widespread (Canham, 2018, Fransman & Yu, 2019).

### Sample

As detailed elsewhere (Ungar et al., 2021), the RYSE advisory committee and gatekeepers (e.g., staff from local non-government organizations) facilitated recruitment. Participant eligibility was determined by: (i) locality (living/attending school/working in RYSE-sites); (ii) age (14–24 years); (iii) English literacy; and (iv) awareness of (negative/positive) O&G industry-related impacts (e.g., witnessed or experienced industry-related layoffs; benefitted from industry-sponsored community investment programs). Eligible participants were invited to recruit peers who fit the eligibility criteria.

Most participants (i.e., 85.7%) self-identified as Black African (see Table 1). Young women were the majority (i.e., 55.7%). Younger adolescents (13–17-year-olds; mean age = 16.13, SD = 1.19) outnumbered older adolescents; only 37.5% were aged 18–24 (mean age = 20.62, SD = 1.53). Most (78.7%) were school attending.

**Table 1:** Summary of participant demographics (*n* = 891)

Variable	Category	Frequency	Percentage
Race	1 = White	109	12.2
	2 = Black	764	85.7
	3 = Coloured	10	1.1
	4 = Indian	6	0.7
	5 = Indigenous	0	0
	6 = Other (specify)	1	0.1
	Missing	1	0.1
Gender	1 = Female	496	55.7
	2 = Male	385	43.2
	3 = Other	3	0.3
	Missing	7	0.8
Age categories	13–17 years	554	62.2
	18–24 years	333	37.5
Are you at school?	1 = Yes	745	83.6
	2 = No	113	12.7
	Missing	33	3.7
Grade	Grade 8	13	1.5
	Grade 9	186	20.9
	Grade 10	188	21.1
	Grade 11	175	19.6
	Grade 12	139	15.6
	Missing	190	21.3

### Data Collection

#### Biographical information

This included race, gender, age, and school attendance.

#### Caregiver monitoring and warmth

Four items from the Parental supervision subscale of the Parenting Scale (Ruchkin et al., 2004) were used to measure parent/caregiver monitoring (e.g., “If living with a parent/caregiver, when you are not home, do they usually know where you are?”). Three items of the Parental Warmth subscale of the Parenting Scale (Ruchkin et al., 2004) were used to assess caregiver warmth (e.g., “Is there a parent/caregiver who shows their love for you?”). Responses were rated on a scale varying from 1 (‘Never’) to 4 (‘Most of the time’).

#### Depression

The Beck Depression Inventory-II (BDI-II; Beck et al., 1996) was used to measure depression symptoms in the preceding 14 days. It has 21 statement sets, each specific to a symptom of a depression with a 4-point (0-3) scale to examine severity. For example: “0 = I get as much pleasure as I ever did from the things I enjoy; 1 = I don’t enjoy things as much as I used to; 2 = I get very little pleasure from the things I used to enjoy; 3 = I can’t get any pleasure from the things I used to enjoy”.

#### Conduct problems

Symptoms of conduct problems are aggression, destruction of property, theft, and other serious rule/law violations (American Psychiatric Association [APA],^ 2013^). These were measured using the 5-item Enactment of Violence Scale (EVS; Geldhof et al., 2014; e.g., “Hit or beat up someone”) and one item about “bullying others”. Participants reported how often they had enacted these behaviours in the past 12 months. Responses were scored as a five-point scale (1 = ‘Never’ to 5 = ‘5+ times’).

#### Data collection procedures

Trained research assistants (RAs) facilitated survey completion, either one-on-one or in small groups (as per participant preference or logistical constraints). Small group survey administration has been previously used in SA resilience studies (Van Rensburg et al., 2019). Some participants worked independently; mostly though, RAs read items aloud before participants self-completed them.

### Statistical Analysis

The descriptive statistics were computed with SPSS 26.0 (IBM Corp, 2020). The Maximum Likelihood Robust (MLR) estimator in Mplus 8.6 (Muthén & Muthén, 2009-2021) was used to test the measurement models of caregiver monitoring and warmth, depression, and conduct problems. Model fit was assessed using the chi-square (χ^2^) value, Standardized Root Mean Residual (SRMR < 0.08) and Root Mean Square Error of Approximation (RMSEA < 0.08), Tucker-Lewis index (TLI > 0.90) and comparative fit index (CFI > 0.90) (Wang & Wang, 2020). We did not include a suitable marker variable (as recommended by Lindell & Whitney, 2001) to check for the possibility of common method variance (CMV), but we used Harman’s single factor test. Although Harman’s test has been criticised (see Cooper et al., 2020), it is regarded as the bare minimum to detect CMV. Therefore, exploratory factor analysis was conducted on the items of all the measures. We examined the unrotated factor solution to determine if there was a single dominant factor that accounted for the majority of variance (>50%). The results showed that a single factor explained 21.84% only of the total variance. Consequently, we did not regard CMV as a threat to the validity of the study.

Latent profile analysis (LPA) in Mplus 8.6 (Muthén & Muthén, 2009-2021) was used to determine distinctive profiles of caregiver monitoring and warmth (Wang & Wang, 2020). Mplus by default imposes local independence across classes, which means that the indicators are constrained to be uncorrelated within each latent class. If a model fits well, then that means that local independence has been achieved. Factor scores for caregiver monitoring and warmth were used as inputs for the LPA. Model fit was assessed using the Akaike information criterion (AIC), Bayesian information criterion (BIC), and sample-size adjusted BIC (ABIC) (Geiser, 2013). We also used the Lo-Mendell-Rubin test (LMR LR), the adjusted Lo-Mendell-Rubin test (ALMR) test, and the bootstrapped likelihood ratio test (BLRT) to determine the optimal number of profiles (Lo et al., 2001, Wang & Wang, 2020). We used the entropy measure to determine how accurately each LPA model partitioned the data into profiles (Ferguson et al., 2020). The entropy of a profile can vary from 0 to 1, with higher values indicating better fit to the data. Point estimates of scale reliability were determined with coefficient Omega (ω), instead of Cronbach’s alpha, since Omega considers the strength of association between items (Hayes & Coutts, 2020). A cut-off score of 0.70 was used.

The automatic Bolck, Croon and Hagenaars (BCH) method (Asparouhov & Muthén, 2014; Bakk & Vermunt, 2016) was used to determine the mean of a distal continuous outcome across latent profiles. Means of the auxiliary variables across the different profiles were determined with the BCH approach to ensure that a shift in the latent profiles did not occur (Asparouhov & Muthén, 2014).

The outcome variables (depression and conduct problem symptoms), gender and age (as covariates), and the BCH weights were used in the USEVARIABLES option in Mplus 8.6 and the BCH weights were used as training variables in the TRAINING option of the VARIABLE command. Two regression models were specified for each distal variable. Gender and age were used to predict the distal variables (depression and conduct problems) through a linear regression model.

## Results

### Latent Profile Analysis

To prepare data for the LPA, confirmatory factor analyses were carried out on the four items which measured caregiver monitoring and three items which measured caregiver warmth. The robust maximum likelihood estimator in Mplus 8.6 was used. The following fit statistics were obtained: χ^2^ = 18.78, *df* = 13, *p* = 0.13, scaling correction factor = 1.36; RMSEA = 0.02 [0.00, 0.04], *p* > 0.05; CFI = 0.99; TLI = 0.99; SRMR = 0.02. The standardized regression coefficients of the sub-scale items were statistically significant and varied from λ = 0.60 to λ = 0.72, mean = 0.64 (caregiver monitoring) and λ = 0.68 to λ = 0.83, mean = 0.78 (caregiver warmth). The correlation between caregiver monitoring and warmth was 0.25 (*p* < 0.001).

**Fig. 1:**
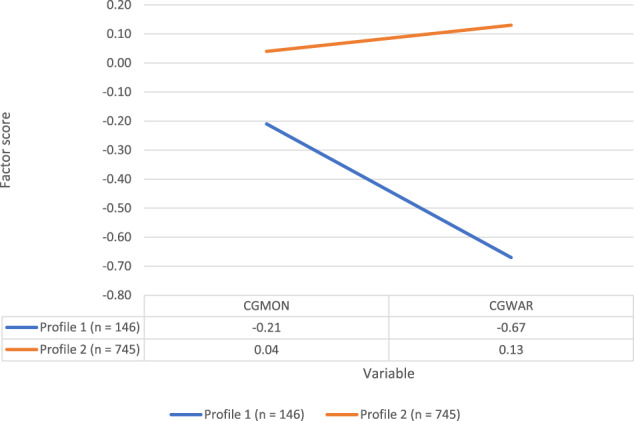
Two latent profiles based on two scales relating to caregiver behavior. Note: CGMON: ‘Caregiver monitoring’; CGWAR: ‘Caregiver warmth’. Higher numbers indicate a higher mean level in caregiver behaviour.

LPA was carried out on participants’ responses to the two caregiver scales, namely caregiver monitoring and caregiver warmth. The random starts of all the LPA models were initially set to 200 with 40 optimisation phases. After acceptable model fit indices were obtained, the random starts were increased 10 times to 2000 with 400 optimisation phases, to ensure findings remain the same. The fit indices are reported in Table 2. The two latent profiles are illustrated in Fig. 1.

**Table 2:** Comparison of Different Latent Profile Analysis Models

Model	AIC	BIC	ABIC	LMR LR test *p*-value	ALMR LR test *p* value	BLRT *p* value
1-profile LPA	1566.94	1586.11	1573.41	–	–	–
2-profile LPA	1052.24	1085.78	1063.55	0.079	0.086	<0.001**
3-profile LPA	593.28	641.20	609.44	0.006**	0.007**	<0.001**

*AIC* Akaike information criterion, *BIC* Bayesian information criterion, *ABIC* adjusted Bayesian information criterion, *LMR LR* Lo-Mendell-Rubin test, *ALMR LR* adjusted Lo-Mendell-Rubin test, *BLRT* bootstrapped likelihood ratio test

**p < 0.01

The fit indices showed significantly better fit for Profile 2 compared with Profile 1 (ΔAIC = 514.70; ΔBIC = 500.33; and ΔABIC = 509.86). The LMR LR (*p* > 0.01), and ALMR (*p* > 0.01) for Profile 2 were not statistically significant. The BLRT for Profile 2 was statistically significant (*p* < 0.01). The fit statistics for Profile 3 showed an improvement from Profile 2. However, only 22 participants were classified in Profile 1. Therefore, it was decided to use the two-profile solution.

Profile 2 had an entropy value of 0.96, suggesting acceptable profile verification (Wang & Wang, 2020). Individuals were profiled with high certainty into the most likely latent profile: 0.96 (Profile 1), and 0.99 (Profile 2). Overall, Profile 1 had lower mean scores and comprised 16.39% of the sample (*n* = 146), whereas Profile 2 consisted of 83.61% (*n* = 745) of the sample.

#### Profile 1: Caregiver monitoring without much warmth (16.39% - *n* = 146)

Individuals in Profile 1 reported a slightly below average level of caregiver monitoring (Mean = −0.18, *SD* = 0.46), and a low level of caregiver warmth (Mean = −0.61, *SD* = 0.36).

#### Profile 2: Substantial caregiver warmth and some monitoring (83.61% - *n* = 745)

Individuals in Profile 2 showed about average levels of caregiver monitoring (Mean = 0.04, *SD* = 0.39), and slightly above average levels of caregiver warmth (Mean = 0.13, *SD* = 0.13).

### Confirmatory Factor Analysis, Descriptive Statistics, Reliabilities, and Correlations

Confirmatory factor analysis (CFA) was conducted to test the factor structures of the measuring instruments for symptoms of depression (BDI) and conduct problems (EVS). The CFA showed acceptable fit: χ^2^ = 579.96 (*df* = 323), *p* < 0.001, RMSEA = 0.03 (0.02, 0.03, *p* > 0.01), CFI = 0.94, TLI = 0.93, SRMR = 0.04. The standardized loadings for the BDI varied from λ = 0.29 (*p* < 0.001) to λ = 0.65 (*p* < 0.001), mean = 0.54. The standardized loadings for the EVS varied from λ = 0.38 (*p* < 0.001) to λ = 0.63 (*p* < 0.001), mean = 0.50. Given that the mean factor loadings were higher than 0.50, highly statistically significant (*p* < 0.001), and factor scores were used in the analyses, we decided to retain all the items of the two measures for further analyses. The descriptive statistics, reliabilities, and Pearson’s correlations of the distal variables are reported in Table 3.

**Table 3:** Descriptive Statistics, Reliabilities, and Pearson’s Correlations of the Distal Variables

Variable	ω	Mean	*SD*	Depression
Depression	0.88	0.68	0.48	–
Conduct problems	0.69	1.37	0.51	0.15*

*p < 0.01

Not shown in Table 3 are the descriptive statistics of the scale scores of caregiver warmth and monitoring. If scale scores rather than factor scores of Profile 2 are analysed, the pattern of higher scores for caregiver warmth (Mean = 3.92, *SD* = 0.19, Minimum = 2.67, Maximum = 4), compared to caregiver monitoring (Mean = 3.29, *SD* = 0.62, Minimum 1, Maximum = 4) is evident. Moreover, in Profile 1, the pattern of lower scores on caregiver warmth (Mean = 2.97, *SD* = 0.51, Minimum 1, Maximum = 3.67) compared to caregiver monitoring (Mean = 3.06, *SD* = 0.72, Minimum 1, Maximum = 4) is evident. The standard deviation of caregiver warmth was low in Profile 2 (and substantially lower than in Profile 1). Lower variation of caregiver warmth existed in Profile 2, and scores started at a higher value than in Profile 1. These descriptive statistics show that caregiver warmth in Profile 1 (compared to Profile 2) varied more, started at a lower level, and had a lower maximum value.

### Latent Profiles and Distal Outcomes

**Fig. 2:**
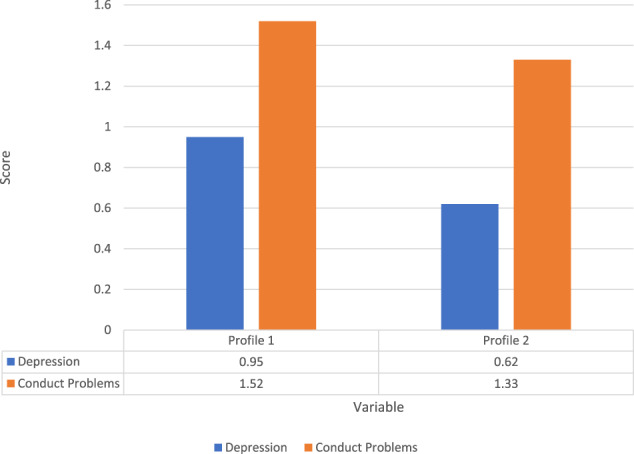
Depression and conduct problems scores in different profiles

Table 4 shows that the participants in Profile 1 scored statistically significantly higher on depression symptoms than those in Profile 2 (χ^2^ = 54.42, *p* < 0.001). Furthermore, participants in Profile 1 scored statistically significantly higher on conduct problem symptoms than those in Profile 2 (χ^2^ = 18.38, *p* < 0.001). Figure 2 illustrates the differences between the depression and conduct problem symptoms of participants in Profiles 1 and 2.

**Table 4:** Equality Tests of Means across Profiles

Depression			Conduct problems		
	Mean	SE		Mean	SE
Profile 1	0.26	0.04	Profile 1	0.17	0.05
Profile 2	−0.05	0.01	Profile 2	−0.03	0.02
Chi-square tests			Chi-square tests		
	χ^2^	*p*		χ^2^	*p*
Overall test	54.42	0.00*	Overall test	18.38	0.00*

*p < 0.01

Gender had a negative effect on the depression score in Profile 1 (estimate = −0.24, *p* < 0.001) and Profile 2 (estimate = −0.11, *p* < 0.001). In both latent profiles, female participants reported higher levels of depression symptoms than male participants. Gender had a positive effect on conduct problems score in Profile 1 (estimate = 0.38, *p* < 0.001) and Profile 2 (estimate = 0.26, *p* < 0.001). Male participants showed higher levels of conduct problem symptoms than female participants in both latent profiles. Age had a negative effect on the conduct problems score in Profile 1 (estimate = −0.06, *p* < 0.01). Younger participants reported higher levels of conduct problem symptoms than older participants in Profile 1.

**Fig. 3:**
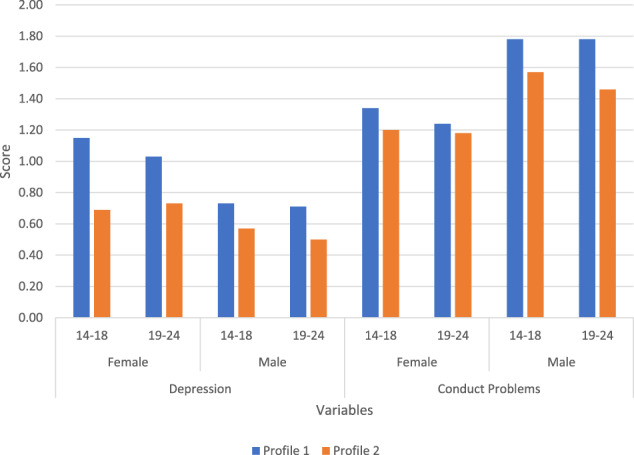
Depression and conduct problems of gender and age groups in different profiles

The mean scores of depression and conduct problem symptoms of female and male participants in different age categories in Profiles 1 and 2 are illustrated in Fig. 3.

## Discussion

Our intention was to investigate distinct profiles of caregiver warmth and monitoring and how these associated with internalising and externalising mental illness symptoms as reported by 891 adolescents (aged 14–24) living in disadvantaged SA communities. The two identified profiles (i.e., Caregiver Monitoring without Much Warmth; Substantial Caregiver Warmth and Some Monitoring) included both monitoring and warm caregiving, albeit to varying extents. While each profile included more or less average levels of caregiver monitoring, only one profile showed considerable levels of caregiver warmth.

The similar levels of caregiver monitoring in both profiles likely relates to the disadvantaged nature of the SA communities in which the RYSE study was conducted. Caregiver monitoring is often prompted by residence in a disadvantaged community and related regular concerns for children’s safety and wellbeing (Pinquart, 2017; Rothenberg et al., 2020c; Shuey & Leventhal, 2019). Although the current study did not investigate adolescents’ contribution to their caregivers’ capacity to monitor them, it is possible that adolescent disclosures about their whereabouts, peer connections, or activities were implicit in the more or less average levels of caregiver monitoring (Kerr & Stattin, 2000). Similarly, it is possible that caregiver monitoring was in response to participants having demonstrated behaviours associated with conduct problems when they were younger (Lansford et al., 2018; Pinquart, 2017).

Caregiver warmth is often sapped by the stressors associated with disadvantaged communities (Conger & Donnellan, 2007; Shuey & Leventhal, 2019). Given that, it is not surprising that 16.39% of participants (i.e., those in the Caregiver Monitoring without Much Warmth profile) reported limited experience of caregiver warmth. Still, caregiver warmth was above average in the second profile (Substantial Caregiver Warmth and Some Monitoring profile), and it was higher than caregiver monitoring. The fact that most of the sample (i.e., 83.61%) reported above average levels of caregiver warmth discourages a priori assumptions that warm caregiving is unlikely in disadvantaged communities (Logan et al., 2019). Instead, it urges attention to the resilience of these stress-exposed caregivers, with special emphasis on better understanding and better supporting the resources that informed their continued capacity to parent positively.

There were clear associations between adolescent mental health and the caregiver monitoring and warmth profile that adolescents belonged to. Adolescents who fit the Caregiver Monitoring without Much Warmth profile reported significantly worse internalising and externalising mental health symptoms than those who fit the second profile. Given that both profiles included almost average levels of monitoring, the level of caregiver warmth appears to be especially crucial to adolescent mental health. This aligns, to some extent, with pre-existing studies that have reported that higher caregiver warmth is associated with better adolescent outcomes (Baumrind, 2005; Logan et al., 2019; Rothenberg et al., 2020a, b, c), also in South African samples of HIV-infected or -affected adolescents (Bhana et al., 2016; Boyes et al., 2019; Casale et al., 2015; Sharer et al., 2015; Shenderovich et al., 2021). Still, none of these prior studies investigated warmth and monitoring combinations and their complex relationships with the mental health of younger and older male and female adolescents. Consequently, our study extends previous understandings of the protective value of caregiver warmth and caregiver monitoring in a disadvantaged community context by showing that about average monitoring with below average warmth enables adolescent mental health significantly less well than about average monitoring combined with above average levels of warmth. Age (i.e., younger or older adolescence) did not alter this association.

As anticipated (Eme, 2007; Salk et al., 2017), higher depression symptom levels were reported by girls and higher levels of conduct problem symptoms by boys. This gender effect applied to both profiles. Given that, neither warm caregiving nor monitoring are apparently sufficient in and of themselves to shift girls’ particular vulnerability to internalising difficulties or boys’ to externalising ones. However, a closer inspection of the findings showed that female and male participants had substantially higher depression scores when they were placed in the Caregiver Monitoring without Much Warmth profile. Likewise, compared with boys in the Substantial Caregiver Warmth and Some Monitoring profile, boys in the Caregiver Monitoring without Much Warmth profile showed slightly higher conduct problem scores. In other words, even though traditional gender effects persisted, these should not detract from the significantly positive association between a combination of considerable levels of warm caregiving and some monitoring, and adolescent mental health.

Although we had no age-related hypotheses, the higher levels of conduct problem symptoms reported by younger adolescents (i.e., 13–17-year-olds) in the Caregiver Monitoring without Much Warmth profile fit with understandings that caregiver monitoring is particularly important in early to middle adolescence (Murphy et al., 2009), possibly because risk behaviours tend to be initiated then (Bhana et al., 2016). Still, the fact that this trend was not reported for younger adolescents in the Substantial Caregiver Warmth and Some Monitoring profile implies that a combination of above average warmth and some monitoring has better protective effects for the mental health of younger adults. Additionally, the current results show the mental health value of above average levels of warm caregiving and some monitoring for younger and older adolescents (18—24-year-olds). Except for Logan et al. (2019), pre-existing studies typically reported such results for adolescents aged 19 or younger (e.g., Bhana et al., 2016; Boyes et al., 2019; Rothenberg et al., 2020a, b, c; Shenderovich et al., 2021). One SA study (Sharer et al., 2015) included adolescents up to age 24, but its focus was solely on supportive parenting and depression symptoms. In short, our study prompts attention to the protective value of warmth combined with some monitoring for younger and older adolescents’ internalising and externalising mental health outcomes.

### Clinical Implications

Taken together, the results encourage clinicians and other service providers to facilitate parenting interventions that go beyond training caregivers to monitor adolescents and/or parent warmly. Instead, they point to the importance of supporting caregivers – particularly those in disadvantaged communities – to understand that these parenting behaviours should be combined, with emphasis on higher levels of warm parenting being preferable to lower ones. Put differently, despite the potential for parental control to moderate the risks of disadvantaged communities to adolescent wellbeing (Hoeve et al., 2009; Pinquart, 2017), moderate monitoring – in combination with more than moderate warmth – has high potential to protect adolescent mental health. Given the importance of parenting warmth to this combination, it is imperative that clinicians enable/sustain caregiver resilience to the stressors that are ubiquitous to disadvantaged communities and known to jeopardise warm parenting (Conger & Donnellan, 2007; Shuey & Leventhal, 2019). In disadvantaged communities, resilience-enabling support for caregivers should transcend informational or emotional support to include practical support (e.g., cash transfers; Eyal & Burns, 2019).

### Limitations

Only urban adolescent insights informed this study’s results. As in Bhana et al. (2016), adding caregivers’ perspectives would have been instructive, as would the perspectives of rural adolescents. In particular, caregivers’ perspectives would have allowed consideration of bidirectional and/or adolescent-driven effects (Lansford et al., 2018; Pinquart, 2017; Rothenberg et al., 2020a, b).

The time periods differed for the symptoms of depression (past 14 days) and conduct problems (past 12 months). Further, we documented cross-sectional insights and so it is unclear whether/how the profiles and related mental health effects will change over time. A longitudinal SA study of parenting effects on adolescent mental health suggested minimal change over time (Shenderovich et al., 2021). Still, like other resilience-enabling resources, parenting factors relate dynamically to adolescent mental health and so longitudinal studies are preferable (Masten, 2014).

## Conclusion

Despite some limitations, this study extends what was known about parenting behaviours and adolescent mental health. It shows, unequivocally, that higher levels of caregiver warmth combined with moderate monitoring have significant protective mental health effects for younger and older adolescents living in disadvantaged communities in South Africa. Previously, older adolescents and African adolescents were under-represented in studies documenting the value of caregiver warmth and monitoring to young people’s mental health. In short, these findings point to the importance of clinicians supporting those who parent adolescents in adversity-challenged communities – also in Africa – to parent warmly and supervise moderately, regardless of the adolescents’ age.

## Data Availability

The data are stored in an institutional repository and can be made available upon reasonable request to the corresponding author.
